# Oxy-Butane Ablation Testing of Thermal Protection Systems Based on Nanomodified Phenolic Resin Matrix Materials

**DOI:** 10.3390/polym15194016

**Published:** 2023-10-07

**Authors:** George Pelin, Cristina Elisabeta Pelin, Adriana Stefan, Violeta Tsakiris, Alexandra Ana Maria Panait, Emil Costea

**Affiliations:** 1 INCAS—National Institute for Aerospace Research “Elie Carafoli”, B-dul Iuliu Maniu 220, 061126 Bucharest, Romania; pelin.george@incas.ro (G.P.); stefan.adriana@incas.ro (A.S.); costea.emil@incas.ro (E.C.); 2 National Institute for Research and Development in Electrical Engineering, 313 Splaiul Unirii, District 3, 030138 Bucharest, Romania; violeta.tsakiris@icpe-ca.ro; 3 Fokker Engineering Romania S.R.L., B-dul Dimitrie Pompeiu 5-7, 020337 Bucharest, Romania; alexandra.panait@fokker.com

**Keywords:** TPS, ablative materials, thermal conductivity, oxy-butane flame test

## Abstract

Two classes of thermal protection systems composed of a carbon-fibre-reinforced (CFRP) layer and an ablative material layer joined with a thermo-resistant ceramic adhesive were developed. The two classes differ in the composition of the ablative material reinforcing compound. In the first class, the ablative material is based on micronic-sized cork granules, and in the second class, the ablative material is reinforced with carbonic felt. For both classes of thermal protection systems, the reinforcement material was impregnated in simple phenolic resin, and nanometric additive, consisting of silicon carbide nanoparticles added in two different weight contents (1 and 2% by weight) relative to the resin. The thermal conductivity for the ablative materials in the thermal protection systems structure was determined. A test facility using oxy-butane flame was developed through which the thermal protection systems developed were tested at extreme temperatures, to simulate some thermal conditions in space applications. The materials were characterised from a morphostructural point of view using optical and scanning electron microscopy after thermal testing. The TPS composed of the carbon-felt-based ablative layer showed improved behaviour compared to the cork-based ablative ones in terms of the temperature increase rate during thermal conductivity testing, mass loss, as well as morphostructural appearance and material erosion after oxy-butane testing. The nSiC-based samples in both sets of TPSs showed improved behaviour compared to the un-filled ones, considering the temperature increase, mass loss, and morphostructure of the eroded material.

## 1. Introduction

As research advances, the discovery of outer space accelerates and technological needs become increasingly complex and interdependent. Ablative materials are at the base of the entire aerospace industry because, as sacrificial materials, they allow the production of propulsion devices or the protection of vehicles and probes during hypersonic flight through a planetary atmosphere. Some nonpolymeric materials have been successfully used as ablatives but, due to their versatility, polymeric ablative materials represent the widest family of sacrificial thermal protection system (TPS) materials [1]. General thermal testing (i.e., DSC, TGA, TMA, etc.) can help to evaluate the behaviour of ablative/TPS materials when exposed to extreme temperature values, but they offer limited information in terms of simulating the real environment conditions. Oxy-acetylene torch tests are one of the most often used to mimic the thermal environmental exposure to which TPSs have to respond [2,3,4,5].

Interest in the aerospace field for nanocomposite materials has grown more and more, due to the excellent properties (mechanically, thermally, electrically, tribologically, etc.) that nanofillers can induce in classic materials [6]. As research and the aerospace industry reach further into outer space, the main problem remains the safe return to Earth of space vehicles due to re-entry into the atmosphere where temperatures reach up to 1200–1500 °C [7,8]. Among the established materials used as thermal protection are carbon preforms impregnated with phenolic resins (PICA—phenolic-impregnated carbon ablator type), epoxy-novolac systems with glass fillers (AVCOAT), and silicon-impregnated ceramic ablative materials (SIRCA—silicone-impregnated reusable ceramic ablator) [9].

There are two distinct classes of thermal protection materials depending on their nature: reusable materials, i.e., carbon–carbon composites, and non-reusable, i.e., ablative materials [10]. Several new lightweight materials alternatives, capable of withstanding extreme conditions, while being profitable from an economic point of view [6,7,8,9,10,11,12,13,14,15,16] have been developed and tested. Thermal protection is an essential factor of structural integrity for a spacecraft vehicle during the re-entry phase. High thermal resistance of the corresponding material together with a limited mass is required against a short intense thermal and mechanical load, leading to a major technical challenge for spacecraft-related research and development [17]. Thermal protection systems—TPSs (so-called thermal shields)—consist of several layers of material joined and applied to the surface of vehicles with the main role of maintaining the temperature inside the vehicle within certain limits during the entire period of re-entry into the atmosphere [18]. Different compositions and architectures of TPSs were developed and tested in the last two decades. The oxidation behaviour of C/SiC composites prepared through chemical vapor infiltration (CVI), at temperatures ranging from room temperature to 1700 °C, was found to be controlled via different mechanisms: below 700 °C, oxidation was controlled via oxygen diffusion through coating cracks; below 1550 °C, it was controlled via the extrinsic diffusion of oxygen through the oxide film on the coating; and up to 1700 °C, oxidation through intrinsic diffusion was found to be dominant [19]. Further analysis [20] regarding the flexural strengths in the combustion atmosphere and weight changes in the air found that different oxidation regimes (i.e., uniform, non-uniform, superficial) become dominant during the temperature range, causing weight changes in air and strength changes in the combustion atmosphere. Preparing C/SiC via standard and low-pressure CVI methods and subjecting them to oxidation tests in dry air from room temperature to 1500 °C concluded that their oxidation behavior could be described by a continuous function over the full temperature range, regardless of how the composites were prepared [21]. Fine control of the microstructure of SiC protective coatings on C/C composites is required for the improvement in both oxidation inhibition and ablation resistance [22]. Adding SiC filler to carbon–carbon composites via powder infiltration techniques led to enhancement in the ablation properties owed to the SiO_2_ formation that prevented heat transfer into the interior of the composites and hindered oxygen diffusion [23]. When subjecting pierced C/C composite nozzles to an oxygen/ethanol hot combustion mixture, the ablation rate was found to be a function of the oxygen/ethanol ratio and could be interpreted by the thermochemical ablation model; for lower ratios, the ablation was limited by chemical kinetics and low ablation rates were observed, while for higher ratios, the ablation was controlled by the diffusion mechanism and high ablation rates were observed [24]. Higher-temperature ablation using the oxy-acetylene flame of C/SiC composites attested the same ablation behaviour modification at different temperature ranges: at 2900 °C, thermal decomposition and oxidation of the SiC matrix were the main ablation mechanism identified, while above 3550 °C, sublimation of carbon fibre and the silicon carbide matrix was the main ablation mechanism identified [25]. The ablation mechanism of 3D orthogonal Cf/SiC composites under oxyacetylene conditions was identified as a combination effect of thermo-oxidation ablation, thermo-physics ablation, and mechanical erosion. Scanning electron microscopy of different ablation regions on the tested surface indicated that the centre region showed sublimation and mechanical erosion, while towards the outer regions, the mechanism was a combination of oxidation and mechanical erosion [26]. Several factors influence the ablative TPS behaviour, from its composition and manufacturing process to the oxygen/ethanol ratio in the used torch. To the best of our knowledge, no study found in the literature uses oxygen and butane gases as an ablation environment. The aim of our study was to obtain thermal protection systems formed by joining a layer of ablative material and a layer of composite material reinforced with carbon fibre, using a ceramic adhesive, and to evaluate its behaviour when tested in oxy-butane torch conditions, to estimate the system capabilities as a suitable candidate for applications in the aerospace and space industry.

## 2. Materials and Methods

The materials used consisted of a commercial ceramic thermo-adhesive Izochit 150 (based on Al_2_O_3_ (minimum 45%) and Fe_2_O_3_ (maximum 1%) and with an operating temperature of up to 1500 °C), purchased from S.C. Prodrefra S.R.L. Brașov, Romania, which combines a carbon fibre material impregnated in phenolic resin (ISOPHEN 215 SM 57%, supplied by S.C. ISOVOLTA S.A., Bucuresti, Romania) [27] and two distinct classes of ablative material, one based on carbon felt [28] and one based on micronic cork granules [10], both impregnated with phenolic resin in simple form and/or nanofilled with nSiC nanoparticles, with 97.5% purity, a specific surface area of 34–40 m^2^/g, and a density of 3.22 g/cm^3^ (purchased from Nanostructured & Amorphous Materials Inc., Los Alamos, NM, USA), for which manufacturing, characterisation, and testing were presented in previous studies [10,27,28].

**Figure 1:**
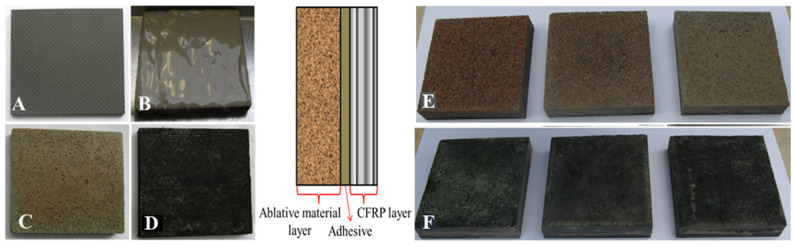
The development procedure for the thermal protection systems: (**A**) PR-CF laminated composite, (**B**) Izochit 150 ceramic adhesive, (**C**) PR-nSiC-cork ablative composite, (**D**) PR-nSiC-felt ablative composite, (**E**) the laminate/adhesive/cork ablative systems developed, and (**F**) the laminate/adhesive/felt ablative systems developed.

1. The thermal protection systems consisted of a layered assembly composed of 3 components:
2. Laminated composite materials (CFRP layer) based on phenolic resin and carbon fibre fabric (named PR-CF);
3. Ceramic adhesive Izochit 150;

Ablative-type materials based on phenolic resin in neat form and with nanometric silicon carbide nanofiller, reinforced by carbon fibre felt (ablative PR-felt) and cork, respectively (ablative PR-cork) (Figure 1).

The development procedure for the thermal protection systems consisted in the development of the carbon-reinforced polymers layer (Figure 1A) and the ablative layers (Figure 1C,D) (following the procedures previously described in [10,27,28] and joining them using a commercial heat-resistant ceramic adhesive (Figure 1B)). The carbon-reinforced polymer layer consisted of 5 layers of carbon fibre impregnated in phenolic resin, made using the thermal hardening process under pressure with a progressive temperature increase from 25 °C to 150 °C, as described in detail in the previously published study [27]. The cork ablative layer and the felt ablative layer were obtained using the same steps of impregnating the ablative preform in simple phenolic resin nano-doped with nSiC, following the temperature curve for reticulation described in previous works [10,28].

To form the TPS structure, the ceramic adhesive was applied between the CFRP and ablative layers, and a constant pressure of 1 kg of force was maintained for 48 h on the entire surface of the TPS, for a uniform and constant-thickness adhesion and to complete the process of hardening the adhesive and fully fixing the components.

Figure 1E,F illustrate the final thermal protection systems obtained at the end of the development process.

Therefore, two categories of systems were developed, differing in the reinforcing preform used in the ablative layer—carbon-felt-based and cork-based. The two systems had three samples having neat and nanofilled phenolic resin as the matrix of the ablative layer. The samples’ nomenclature and composition are presented in the Table 1.

## 3. Testing and Characterisation

### 3.1. Thermal Conductivity Measurement

Before thermal testing of the systems, the ablative layer thermal conductivity was measured using the equipment LFA 447 NanoFlash, NEZSCH, at temperatures ranging from room temperature to 300 °C (five analyses were performed at each temperature step), according to the EN 821-2 standard [29].

### 3.2. Oxy-Butane Flame Testing Assembly Development

The oxy-butane testing aimed to reproduce the thermal conditions during the re-entry phase.

**Figure 2:**
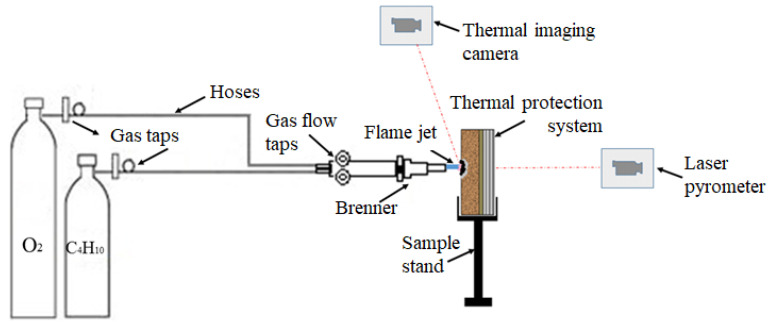
Schematic representation of the oxy-butane flame testing facility components.

The scheme of the oxy-butane flame jet facility is presented in Figure 2. The facility was designed in the Materials and Tribology Unit, following the specific requests to obtain the temperature values of 1200–1300 °C, reached during space applications such as the re-entry process.

**Figure 3:**
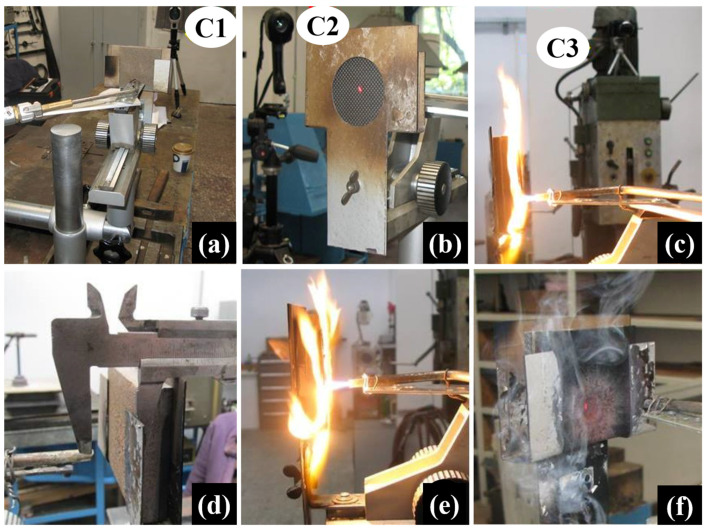
Upper side—video/data recording systems of the oxy-butane flame test facility assembly: (**a**) front view of the test assembly C1—laser pyrometer that records the temperature behind the sample, (**b**) back view of the test assembly C2—thermal imaging camera that records the temperature on the surface of the sample exposed to the flame, (**c**) side view during testing with oxy-butane flame C3—video camera that records throughout the test. Bottom side—stages of the procedure: (**d**) distance measurement between the sample and Brenner tip, (**e**) image of the action of torch on the sample during testing, and (**f**) image of the sample immediately after the torch action was stopped.

The facility consists of two gas cylinders (butane and oxygen) connected to a GCE RK-20 2A Brenner, with a 1–2 mm diameter, that ensures a gas flow value of 160 L/h at 2.5 bar. The facility includes a thermal imaging camera (Figure 3b—FLIR T1020) that captures the temperature on the contact surface of the thermal protection system during the action of the oxy-butane flame and a laser pyrometer (Figure 3a—Cole-Parmer Infrared Thermometer) that records the temperature on the opposite surface of the thermal protection system in order to observe the thermal transfer temperature during thermal testing. The thermal test is recorded on video using a Canon G7 video camera (Figure 3c).

After the sample was mounted into the sample holder, the distance between the sample and the Brenner burner tip was set to 20 mm, using callipers as Figure 3d illustrates. The torch action was set to 120 s and the initially measured distance was precisely maintained for the whole duration of the test (Figure 3e). Figure 3f shows one of the TPS samples immediately after the oxy-butane torch stopped its action, and the infrared projection point of the thermal imagining camera (C2) can be observed. The oxy-butane flame performed its action on a point-like area as it can be seen in Figure 3f, with the images in Figure 3c,e illustrating the radial dispersion of the flame around the action point.

### 3.3. Optical Microscopy

The surface of the ablative layer that was in contact with the oxy-butane flame was visualised using the Meiji 8520 optical microscope, equipped with the Infinite Analyze-Lumenera Corporation video camera for electronic image capture and recording, at 25× magnification level.

### 3.4. Scanning Electron Microscopy

For the analysis of nanofiller-based ablative components of the system scanning electron microscope, QUANTA FEI 250 equipment was used, with a field emission gun with a resolution of 1.0 nm and an energy-dispersive X-ray spectrometer (EDS) with a resolution of 133 eV.

SEM analysis was recorded on the surface of the ablative material in the area of action of the oxy-butane flame flow.

## 4. Results and Discussion

### 4.1. Thermal Conductivity

The thermal conductivity was performed on the surface with the ablative part of the thermal protection system for all individual samples. The measurement of the thermal conductivity of the materials was carried out in the temperature range of 25 to 95 °C.

**Figure 4:**
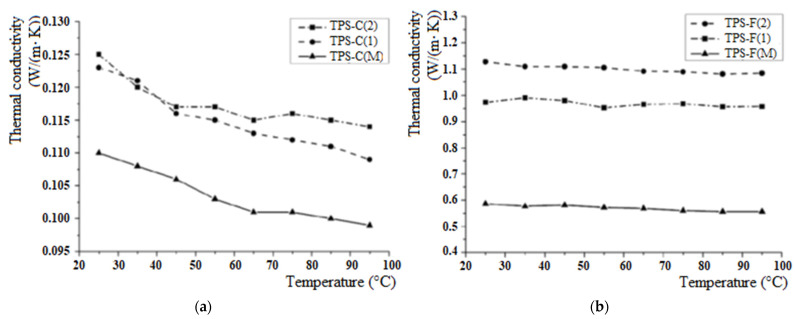
The evolution of the thermal conductivity of the thermal protection systems depending on the temperature: (**a**) TPS cork-based and (**b**) TPS felt-based.

Figure 4 illustrates the evolution of thermal conductivity of thermal protection systems in the temperature range of 25–95 °C. It can be observed that the mass percentage of silicon carbide of nanometric dimensions in the composition of the ablative layer leads to an increase in the thermal conductivity of the system, while the conductivity of all samples decreases with increasing temperature. This is an expected effect because, besides the presence of nSiC, which is a compound with high thermal conductivity [30], the nanocomposite materials also have a higher density compared to the control sample. Furthermore, with the increase in nSiC content, the areas with high thermal conductivity increase, which leads to the enhancement in the thermal conductivity of the ablative layers.

There are major differences in thermal conductivity between the two sets of TPSs. These differences are due to the ablative layer contained in each individual system and their different thermal conductivity range. The cork ablative layer has low thermal conductivity, due to its wood-type nature (approximately 0.05 W/mK [31]), representing one of the oldest insulation materials used, while carbon/phenolic-type materials are very efficient from the ablative point of view, but have high density [32]. On the other side, the higher thermal conductivity of the carbon-felt-ablative-layer-based TPS is generated by the elevated thermal conductivity of carbon felt, together with the continuous morphology of this preform compared to the granulated cork form.

### 4.2. Thermal Testing of Thermal Protection Systems

The oxy-butane flame facility was designed within this study and the test consisted of applying the flame perpendicular to the surface of the thermal protection system, on the side with the ablative material layer, and maintaining the flame action, which induces temperatures of over 1300 °C for 120 s (2 min). This dwell time was chosen based on the existing data in the literature, which states that this is the estimated duration of the “thermal boom” of the stage of re-entry into the Earth’s atmosphere of a vehicle returning from outer space [33]. At the end of the test, the samples were removed from the holder and allowed to cool naturally to room temperature.

**Figure 5:**
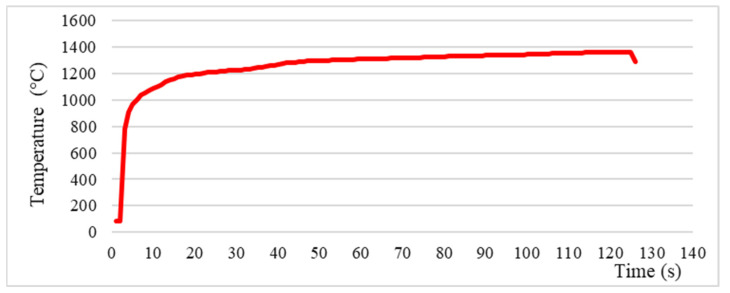
Evolution of the temperature on the surface of the sample during the 120 s of exposure in the oxy-butane flame.

Figure 5 shows the temperature–time curve recorded by the thermal imaging camera on the TPS surface where the oxy-butane flame performs its action, for the entire period of the test.

**Figure 6:**
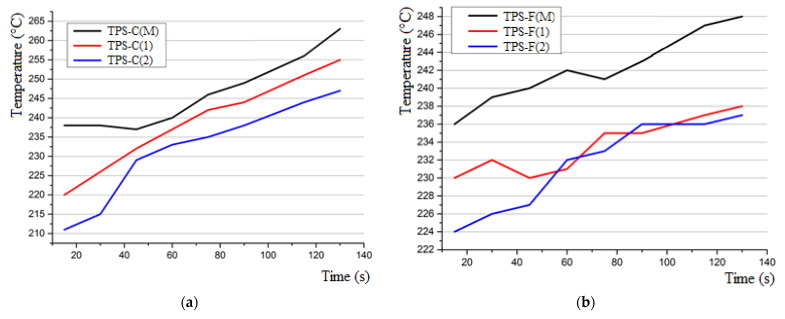
Temperature evolution on the surface of the CFRP layer in the backside of the thermal protection system ((**a**) TPS cork-based and (**b**) TPS felt-based) during the 120 s of exposure to the oxy-butane flame.

Figure 6 shows the temperature–time curves recorded by the laser pyrometer on the TPS surface behind the area where the oxy-butane flame performs its action, for the entire period of the test. It can be observed that the samples that previously exhibited higher thermal conductivity (i.e., the nSiC-based samples versus control samples, and carbon-felt-based samples versus cork-based ones) show a lower temperature increase during the 2 min testing under the torque action. This would be explained by the fact that in higher-thermal-conductivity materials, the energy is more evenly distributed and more effectively dissipated in the whole volume of the sample, resulting in a more efficient “response” from the material to the thermal stimulus and a slower heating rate.

Therefore, it is observed that in both sets of TPS samples that have the ablative layer with the addition of nSiC, the recorded temperature is lower than the one recorded in the case of the systems that do not have nanofiller as part of the ablative layer, for both sets of the developed TPS.

This may suggest that nSiC can act as a thermal shield, by increasing the thermal resistance of the ablative material, which is thus consumed more slowly than that without nSiC addition, thus decreasing heat propagation (thermal transfer) to the layers behind the layer ablative.

**Figure 7:**
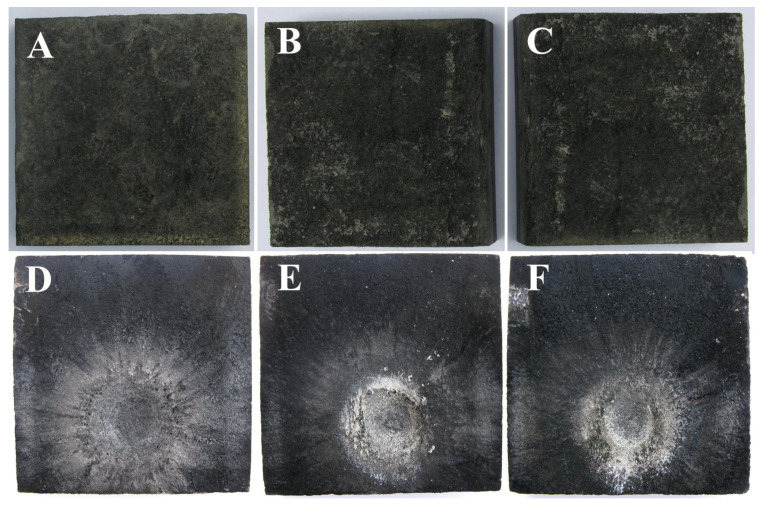
TPS—carbon felt ablative layer, before and after testing in the oxy-butane flame: TPS-F(M) before (**A**) and after (**D**); TPS-F(1) before (**B**) and after (**E**); and TPS-F(2) before (**C**) and after (**F**).

**Figure 8:**
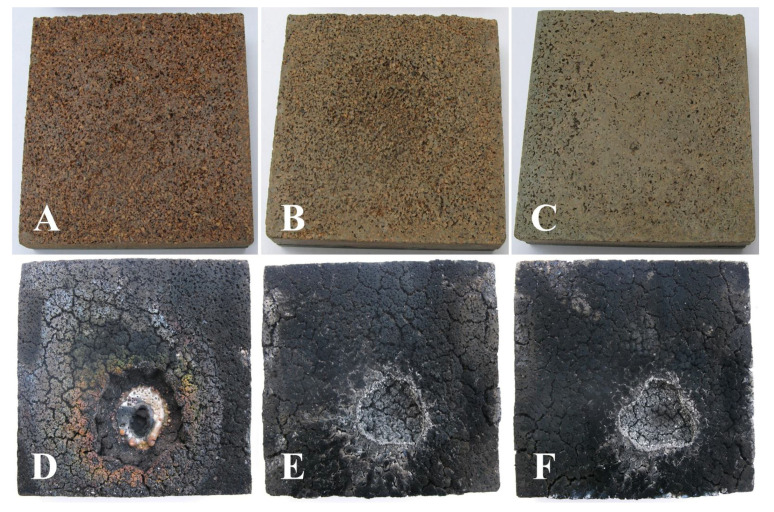
TPS—cork ablative layer, before and after testing in the oxy-butane flame: TPS-C(M) before (**A**) and after (**D**); TPS-C(1) before (**B**) and after (**E**); and TPS-C(2) before (**C**) and after (**F**).

Also, the set of TPS samples that have an ablative layer based on carbon felt have lower temperature values than the set of TPS samples that have an ablative layer made of cork, a fact due to the superior thermal properties of carbon felt (Figure 7) that shows lower erosion of the ablative layer compared to the one in the cork (Figure 8). The control samples were the most intensely penetrated by the action of the flame, suffering damage both on larger areas and deeper in the material, with the systems with the ablative layer based on cork presenting the total penetration of the ablative layer, up to the PR/CF composite layer.

It is important to mention that the ceramic adhesive successfully withstood the action of the oxy-butane flame on the systems, indicating both the high protection that the ablative layer is able to provide to the systems, as well as high thermal resistance of the adhesive that managed to sustain the joining of the components that form the systems.

#### Gravimetric Loss Analysis after Testing

Both sets of TPSs were weighed on an analytical balance before and after the oxy-butane flame test to observe the mass loss after the test.

As can be seen from the pictures of the samples tested in the flame, the mass loss of the ablative component occurs on the surface that comes into contact with the flame and radially around it (Figure 7 and Figure 8).

**Figure 9:**
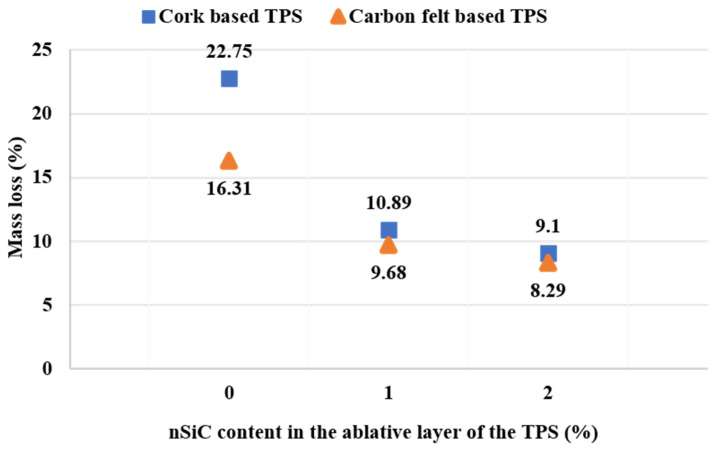
Mass loss of thermal protection systems after the oxy-butane flame test for the two sets of TPSs.

It is thus observed that the control samples suffer the greatest mass loss (16–23%) with the penetration of the simple ablative layer (Figure 9).

In the case of the nanocomposite samples, the differences compared to the control samples are significant; they show mass losses of approximately 8–10%, with the losses becoming smaller with the increase in the percentage of nanofiller (Figure 9). The gravimetric analysis is in accordance with the visual inspection of the samples, which clearly shows how the control samples were the most intensely penetrated by the action of the flame, in the case of cork-based samples, presenting the total penetration of ablative layer, up to the PR/CF composite layer. Previous studies that were conducted on the ablative layers only [10,28] presented TG-DSC analysis on the two sets of ablative layers, with the results being in accordance with the present gravimetric analysis data trend and the nSiC presence contributing to increased thermal resistance in terms of material consumption at high temperature.

### 4.3. Analysis of the Eroded Surface through Optical Microscopy

Optical microscopy analyses help to observe surface cracks and the general appearance of the sample on the ablative material layer surface that was subjected to the action of the flame.

**Figure 10:**
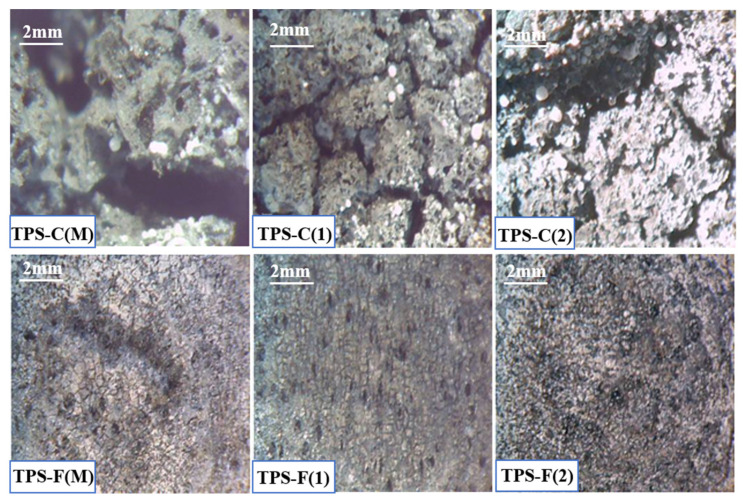
Optical microscopy images of TPS samples after the oxy-butane flame test.

Figure 10 illustrates the area of direct action of the oxy-butane flame, observing the deep mechanical erosion of the ablative material and the pronounced cracking of the exposed surface.

TPSs that have in their composition the ablative part based on cork show accentuated areas of thermal degradation with cracks and penetrations of the layer compared to TPSs that have in their composition the ablative part based on carbon felt. It can be observed that in the control sample with cork-based ablative, the material was fully penetrated, with the exposure to the oxy-butane flame generating deep cracks, that penetrate down to the ceramic adhesive layer. As the nSiC content increases, the extent of the crack tends to decrease, observing narrower crack-related damage.

Carbon-felt-based TPSs show a less eroded surface of the samples, with small-sized cracks and no full penetration of the ablative layer. This can be attributed to the high thermal conductivity of carbon felt previously discussed, which manages to distribute and dissipate the energy more efficiently in the whole volume of the sample, resulting in less degradation suffered by the exposed area. Also in this case, nSiC manages to improve the morphology of the materials from the points of view of erosion and damage to the surface and in-depth. The presence and dispersion of silicon nanocarbide are further investigated in the next section through SEM analyses.

### 4.4. Analysis of the Eroded Surface through Scanning Electron Microscopy (SEM)

**Figure 11:**
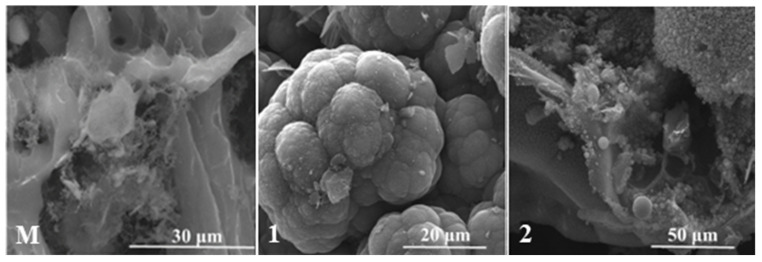
SEM images of the TPS with cork-based ablative layer.

The SEM images illustrate the deep carbonisation of the material, with areas that present the appearance of burnt wood in the case of TPSs with cork-based ablative, with a clear appearance of this damage in the cork-based control sample (Figure 11).

**Figure 12:**
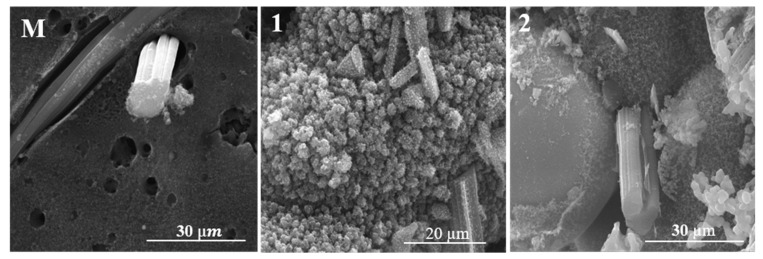
SEM images of the TPS with felt-based ablative layer.

In the case of the composites with nSiC in their composition (Figure 11(1,2) and Figure 12(1,2)), the morphology resulting from the flame burning of the materials is very different from that of the control sample (Figure 11(M) and Figure 12(M)). There are areas in which agglutinated spheres can be observed with white zones around them. This morphology could be attributed to the silicon carbide oxidation mechanism that occurred during oxy-butane torch action. Under oxidative environments at high enough temperatures, solid SiC undergoes reactions that transform part of it into SiO_2_ and CO [34,35]. When oxidation occurs at an oxygen pressure of less than 1 bar, the SiO_2_ formed vaporises after its formation, leading to mass loss; when it occurs at an oxygen pressure of around 1 bar, SiO_2_ deposits over the surface of SiC, leading to an increase in mass (or rather a smaller mass loss in our case) [34]. The mentioned areas seem to be composed of nSiC agglutinated into grains, but as some parts of SiC oxidised into SiO_2_ with a solid form following oxidation, the latter one manages to protect the nSiC nanoparticles on which it is deposited from further oxidation [35]. This could be due to the mechanical erosion by the high-speed oxy-butane torch that quickly smashed the SiC nanoparticles [35,36].

**Figure 13:**
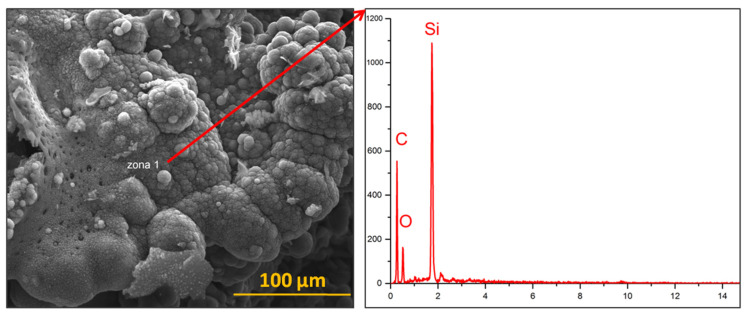
EDS analysis of a TPS-C(2) sample area.

The EDS analysis of a TPS-C(2) area with the mentioned nSiC layer appearance (Figure 13) confirms the majority presence of the Si element, followed by the C element (resulting from both nSiC and the carbonised areas) and a significant quantity of O element, sustaining the hypothesis that the agglutinated nSiC nanoparticles are present and covered by a protective layer of SiO_2_.

**Figure 14:**
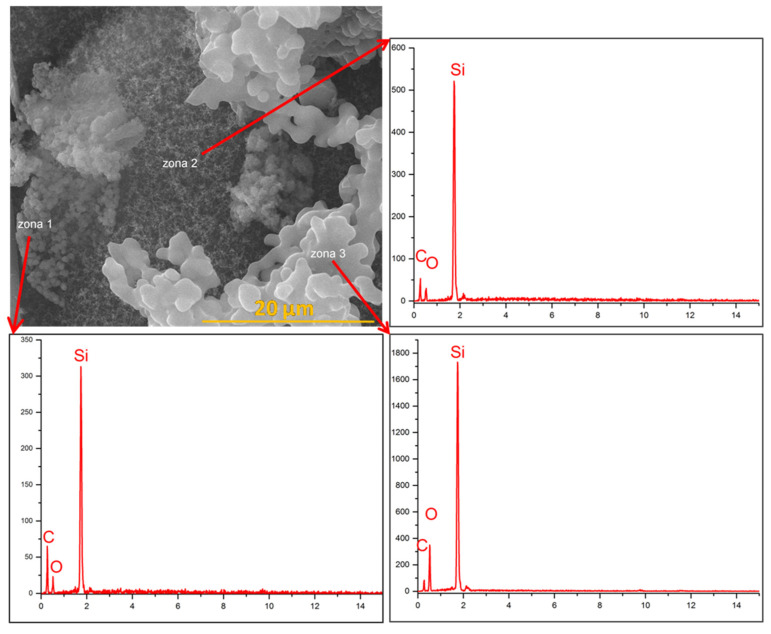
EDS analysis of a TPS-F(2) sample.

The EDS analysis of an area of the TPS-F(2) sample (Figure 14), which presents 3 zones of different morphology, illustrates the presence of the Si element as the majority in all 3 zones, followed by C and O elements. In zone 3, which illustrates the spherical agglutinated grains with white edges, EDS analysis shows a higher O content compared with the other two zones, exhibiting the C element content. This confirms, as in the case of cork-based materials, the hypothesis that these areas observed in the SEM micrographs are indeed agglutinated nSiC nanoparticles that were covered by the protective layer of SiO_2_ that was deposited on its surface as a consequence of the SiC oxidation mechanism.

SEM and EDS together illustrate that following the SiC reactions under a high-erosion oxidative environment, the solid SiO_2_ resulting from the reaction mechanism managed to successfully protect the SiC nanoparticles whether they were agglutinated or more dispersed on the visualised areas.

The remaining protected nSiC distributed in the entire ablative structure manages to efficiently distribute and dissipate the energy during thermal testing and improve their behaviour when subjected to extreme temperatures.

The visual inspection together with morphostructural analyses through optical and electronic microscopy and elemental analysis via EDS found that the main ablation mechanism during oxy-butane torch testing was a combination of mechanical erosion and oxidation of the surface.

## 5. Conclusions

The behaviour of two classes of thermal protection systems during the subjection to oxy-butane torch was studied within this paper. The TPSs were composed of a CFRP (carbon-fibre-reinforced polymer) layer and an ablative material layer (one based on cork and one based on carbon felt, impregnated in simple phenolic resin and 1 and 2% nSiC added) joined with a thermo-resistant ceramic adhesive.

The experimental plan included, besides the oxy-butane torch testing for 120 s, thermal conductivity measurements of the ablative, gravimetrical analysis before and after testing, optical microscopy, and scanning electron microscopy with EDS.

The results led to two major observations. The TPSs composed of a carbon-felt-based ablative layer showed improved behaviour compared to the cork-based ablative ones in terms of the temperature increase rate during thermal conductivity testing, mass loss, as well as morphostructural appearance and material erosion after oxy-butane testing. The nSiC-based samples in both sets of TPSs also showed this improved behaviour, considering the temperature increase, mass loss, and morphostructure of the eroded material. This could be attributed on one side to the higher thermal conductivity of these components that managed to distribute and dissipate more efficiently the energy in the volume of the samples. On the other side, the continuous morphology of the carbon preform (compared to the granulated cork form) and the uniformly distributed silicon carbide nanoparticles into the ablative contribute to a more efficient response of the material to the thermal stimulus, with lower heating rates and less damaged erosion areas (both in-depth as well as on the surface). Besides the thermal conductivity and morphology-related factors, the oxidation mechanism of SiC nanoparticles was observed through SEM and EDS analysis. The correlation between the elemental analysis and micrographs confirms that some parts of the SiC nanoparticles oxidised into solid SiO_2_, which deposited over the nSiC nanoparticles, protecting them from further oxidation, observed through the white zones on the spherical grains that presented high contents of Si and O elements. The microstructure inspections together with the evaluation of thermal properties found that the main ablation mechanism during oxy-butane torch testing was a combination of mechanical erosion and oxidation of the surface, with the higher nSiC content managing to protect the ablative via two routes: first by more efficiently distributing and dissipating the energy in the sample volume, and second by its behaviour during oxidation that formed a SiO_2_ layer that protected the SiC nanoparticles from further oxidation, consequently protecting the ablative microstructure.

The adhesive successfully withstood the action of the oxy-butane flame on the systems, confirming both the high protection that the ablative layer is able to provide to the systems, as well as high thermal resistance of the adhesive that managed to sustain the joining of the components that form the systems.

Considering the corroboration of all experimental data, it could be concluded that a TPS composed of an nSiC-doped carbon fibre felt phenolic ablative layer and a CFRP joined by a ceramic adhesive could act as a promising candidate for aerospace and space applications where high thermal resistance in oxygen-rich environments is a requirement.

## Figures and Tables

**Table 1:** The samples nomenclature and composition of the thermal protection systems.

Sample Nomenclature	Ablative Layer Preforms	Ablative Layer Matrix	Abbreviation
PR-CF/Adhesive/PR-felt	Carbon fibre felt	Neat phenolic resin (PR)	TPS-F(M)
PR-CF/Adhesive/PR + 1%nSiC-felt	Carbon fibre felt	Phenolic resin and 1% by weight nSiC	TPS-F(1)
PR-CF/Adhesive/PR + 2%nSiC-felt	Carbon fibre felt	Phenolic resin and 2% by weight nSiC	TPS-F(2)
PR-CF/Adhesive/PR-cork	Cork particle	Neat phenolic resin (PR)	TPS-C(M)
PR-CF/Adhesive/PR + 1%nSiC-cork	Cork particle	Phenolic resin and 1% by weight nSiC	TPS-C(1)
PR-CF/Adhesive/PR + 2%nSiC-cork	Cork particle	Phenolic resin and 2% by weight nSiC	TPS-C(2)

## Data Availability

Data available on request.

## References

[1] Rallini M., Natali M., Torre T., Yan Q.-L., He G.-Q., Liu P.-J., Gozin M. (2019). Chapter 14—An Introduction to Ablative Materials and High-Temperature Testing Protocols. Micro and Nano Technologies, Nanomaterials in Rocket Propulsion Systems.

[2] Kumar V. (2017). Ionic-liquid-assisted three-dimensional caged silica ablative nanocomposites. J. Appl. Polym. Sci..

[3] Marra F., Pulci G., Tirilló J., Bartuli C., Valente T. (2011). Numerical Simulation of Oxy-Acetylene Testing Procedure of Ablative Materials for Re-Entry Space Vehicles, Proceedings of the Institution of Mechanical Engineers. Part L J. Mater. Des. Appl..

[4] Arnaud E., Halm D., Bertheau D., Beaudet J. Ablation performances of carbon composite based on different resins under severe aero-thermal flux, ECCM18. Proceedings of the 18th European Conference on Composite Materials.

[5] Beaudet J., Cormier J., Dragon A., Rollin M., Benoi G. (2011). Ablation Properties of C Fibers and SiC Fibers Reinforced Glass Ceramic Matrix Composites upon Oxyacetylene Torch Exposure. Mater. Sci. Appl..

[6] Pelin G. (2017). Materiale Compozite Avansate. Ph.D. Thesis.

[7] Jenkins D.R. (2001). Space Shuttle: The History of the National Space Transportation System the First 100 Missions.

[8] Pirolini A. Materials Used in Space Shuttle Thermal Protection Systems, AZO Materials. https://www.azom.com/article.aspx?ArticleID=11443.

[9] Stackpoole M., Thornton J., Fan W. Ongoing TPS Development at NASA Ames Research Center. Proceedings of the CRASTE Conference Commercial and Government Responsive Access to Space Technology Exchange.

[10] Pelin G., Pelin C.-E., Stefan A., Dinca I., Andronescu E., Oprea O., Ficai D., Trusca R. (2018). Development and properties of advanced composites based on cork and nanometric silicon carbide-filled phenolic resin. Bull. Mater. Sci..

[11] Paixão S., Peixoto C., Reinas M., Carvalho J. (2022). RETALT_TPS design and manufacturing. CEAS Space J..

[12] Dunn B.D. (2017). Materials and Processes for Spacecraft and High Reliability Applications. Aeronaut. J..

[13] Triantou K.I., Mergia K., Perez B., Florez S., Stefan A., Ban C., Pelin G., Ionescu G., Zuber C., Fischer W.P.P. (2017). Thermal shock performance of carbon-bonded carbon fiber composite and ceramic matrix composite joints for thermal protection re-entry applications. Compos. Part B.

[14] Tong Y., Bai S., Liang X., Qin Q.H., Zhai J. (2016). Reactive melt infiltration fabrication of C/C-SiC composite: Wetting and infiltration. Ceram. Int..

[15] Reichert F., Pérez-Mas A.M., Barreda D., Blanco C., Santamaria R., Kuttner C., Fery A., Langhof N., Krenkel W. (2017). Influence of the carbonization temperature on the mechanical properties of thermoplastic polymer derived C/C-SiC composites. J. Eur. Ceram. Soc..

[16] Kumar S., Bablu M., Ranjan A., Manocha L.M., Prasad N.E. (2017). Fabrication of 2D C/C-SiC composites using PIP based hybrid process and investigation of mechanical properties degradation under cyclic heating. Ceram. Int..

[17] Glass D.E. Ceramic Matrix Composite (CMC) Thermal Protection Systems (TPS) and Hot Structures for Hypersonic Vehicles. Proceedings of the 15th AIAA Space Planes and Hypersonic Systems and Technologies Conference.

[18] Pelin C.-E., Pelin G., Ilina S., Dragomirescu A., Cristea G., Stefan A. (2021). Properties of Ablative Composites Based on Bismaleimide Resin Reinforced with Graphite Felt. U.P.B. Sci. Bull. Ser. B.

[19] Cheng L.F., Xu Y.D., Zhang L.T., Yin X.W. (1999). Oxidation behavior of carbon–carbon composites with a three-layer coating from room temperature to 1700 °C. Carbon.

[20] Cheng L.F., Xu Y.D., Zhang L.T., Yin X.W. (2000). Oxidation behavior of three dimensional C/SiC composites in air and combustion gas environments. Carbon.

[21] Cheng L.F., Xu Y.D., Zhang L.T., Yin X.W. (2001). Effect of carbon interlayer on oxidation behavior of C/SiC composites with a coating from room temperature to 1500 °C. Mater. Sci. Eng. A.

[22] Lee Y.J., Joo H.J. (2004). Ablation characteristics of carbon fiber reinforced carbon (CFRC) composites in the presence of silicon carbide (SiC) coating. Surf. Coat. Technol..

[23] Tang S.F., Deng J.Y., Liu W.C., Yang K. (2006). Mechanical and ablation properties of 2D-carbon/carbon composites pre-infiltrated with a SiC filler. Carbon.

[24] Chen B., Zhang L.T., Cheng L.F., Luan X.G. (2009). Ablation of Pierced C/C Composite Nozzles in an Oxygen/Ethanol Combustion Gas Generator. Carbon.

[25] Chen Z., Fang D., Yan B. (2008). Comparison of Morphology and Microstructure of Ablation Centre of C/SiC Composites by Oxy-Acetylene Torch at 2900 °C and 3550 °C. Corros. Sci..

[26] Yan B., Chen Z.F., Li C., Fand D., Zhang Y., Wang L. (2008). Ablation morphology and microstructure of 3D Orthogonal Cf/SiC composites prepared by PIP. Sci. Eng. Compos. Mater..

[27] Pelin G., Pelin C.-E., Ștefan A., Dincă I., Andronescu E., Ficai A., Truşcă R. (2017). Mechanical and tribological properties of nanofilled phenolic matrix laminated composites. Mater. Technol..

[28] Pelin G., Andronescu E., Pelin C.-E., Oprea O., Ficai A. (2016). Ablative type Composites Based on Phenolic Resin/Nanosilicon Carbide Matrix Reinforced by Carbon Fiber Felt. Rom. J. Mater..

[29] (1997). Advanced Technical Ceramics—Monolithic Ceramics—Thermo-Physical Properties—Part 2: Determination of Thermal Diffusivity by the Laser Flash (or Heat Pulse) Method.

[30] Price R.J. (1973). Thermal Conductivity of Neutron-Irradiated Pyrolytic β-Silicon Carbide. J. Nucl. Mater..

[31] Shawyer M., Medina Pizzali A.F. (2003). The use of ice on small fishing vessels. FAO Fish. Tech. Pap..

[32] Federal Aviation Administration, Designees and Delegations Guide: Section 4.1.7. Returning from Space: Re-Entry, Federal Aviation Administration. https://www.faa.gov/about/office_org/headquarters_offices/avs/offices/aam/cami/library/online_libraries/aerospace_medicine/tutorial/section3/spacecraft_design.

[33] Di Benedetto A.T., Nicolais L., Watanabe R. Composite materials. Proceedings of the Symposium A4 on Composite Materials of the International Conference on Advanced Materials-ICAM 91.

[34] Roy J., Chandra S., Das S., Maitra S. (2014). Oxidation behaviour of silicon carbide—A review. Rev. Adv. Mater. Sci..

[35] Wang Y., Chena Z., Yu S. (2016). Ablation behavior and mechanism analysis of C/SiC composites. J. Mater. Res. Technol..

[36] Yan B., Chen Z.F., Zhu J., Zhang J., Jiang Y. (2009). Effects of ablation at different regions in three-dimensional orthogonal C/SiC composites ablated by oxyacetylene torch at 1800 °C. J. Mater. Process. Technol..

